# Predictive *MGMT* status in a homogeneous cohort of *IDH* wildtype glioblastoma patients

**DOI:** 10.1186/s40478-019-0745-z

**Published:** 2019-06-05

**Authors:** Josefine Radke, Arend Koch, Fabienne Pritsch, Elisa Schumann, Martin Misch, Claudia Hempt, Klaus Lenz, Franziska Löbel, Fabienne Paschereit, Frank L. Heppner, Peter Vajkoczy, Randi Koll, Julia Onken

**Affiliations:** 1 Department of Neuropathology, Charité - Universitätsmedizin Berlin, corporate member of Freie Universität Berlin, Humboldt-Universität zu Berlin, and Berlin Institute of Health, Charitéplatz 1 (Virchowweg 15), 10117 Berlin, Germany; 2 German Cancer Consortium (DKTK), Heidelberg, Germany, Partner Site Charité Berlin, Berlin, Germany; 3 Department of Neurosurgery, Charité - Universitätsmedizin Berlin, corporate member of Freie Universität Berlin, Humboldt-Universität zu Berlin, and Berlin Institute of Health, Charitéplatz 1, 10117 Berlin, Germany; 4 Charité - Universitätsmedizin Berlin, corporate member of Freie Universität Berlin, Humboldt-Universität zu Berlin, and Berlin Institute of Health, Institute of Biometrics and Clinical Epidemiology, Berlin, Germany; 5 Cluster of Excellence, NeuroCure, Charitéplatz 1, 10117 Berlin, Germany; 6 BSIO Berlin School of Integrative Oncology, University Medicine Charité, 13353 Berlin, Germany; 7 Berlin Institute of Health (BIH), 10178 Berlin, Germany; 8 German Center for Neurodegenerative Diseases (DZNE) Berlin, 10117 Berlin, Germany

**Keywords:** Glioblastoma, Temozolomide (TMZ), O(6)-Methylguanine-DNA methyltransferase (MGMT), Methylation specific PCR (MSP), Pyrosequencing (PSQ), IDH (isocitrate dehydrogenase)

## Abstract

Methylation of the O(6)-Methylguanine-DNA methyltransferase (*MGMT*) promoter is predictive for treatment response in glioblastoma patients. However, precise predictive cutoff values to distinguish “*MGMT* methylated” from “*MGMT* unmethylated” patients remain highly debated in terms of pyrosequencing (PSQ) analysis. We retrospectively analyzed a clinically and molecularly very well-characterized cohort of 111 *IDH* wildtype glioblastoma patients, who underwent gross total tumor resection and received standard Stupp treatment. Detailed clinical parameters were obtained. Predictive cutoff values for *MGMT* promoter methylation were determined using ROC curve analysis and survival curve comparison using Log-rank (Mantel-Cox) test. *MGMT* status was analyzed using pyrosequencing (PSQ), semi-quantitative methylation specific PCR (sqMSP) and direct bisulfite sequencing (dBiSeq). Highly methylated (> 20%) *MGMT* correlated with significantly improved progression-free survival (PFS) and overall survival (OS) in our cohort. Median PFS was 7.2 months in the unmethylated group (UM, < 10% mean methylation), 10.4 months in the low methylated group (LM, 10-20% mean methylation) and 19.83 months in the highly methylated group (HM, > 20% mean methylation). Median OS was 13.4 months for UM, 17.9 months for LM and 29.93 months for HM. Within the LM group, correlation of PSQ and sqMSP or dBiSeq was only conclusive in 51.5% of our cases. ROC curve analysis revealed superior test precision for survival if additional sqMSP results were considered (AUC = 0.76) compared to PSQ (cutoff 10%) alone (AUC = 0.67). We therefore challenge the widely used, strict PSQ cutoff at 10% which might not fully reflect the clinical response to alkylating agents and suggest applying a second method for *MGMT* testing (e.g. MSP) to confirm PSQ results for patients with LM *MGMT* levels if therapeutically relevant.

**Electronic supplementary material:**

The online version of this article (10.1186/s40478-019-0745-z) contains supplementary material, which is available to authorized users.

## Introduction

Glioblastoma (GBM) is the most common and most aggressive primary brain tumor. The histological examination of neurosurgical tumor specimens as well as the immmunohistochemical or molecular determination of the *IDH1/2* status remain the gold standard for diagnosis of GBM [13]. Despite aggressive therapy, the survival of patients with GBM is approximately 15-17 months [21]. The current standard GBM therapy usually consists of neurosurgical resection, radiotherapy and additional chemotherapy with temozolomide (TMZ), an alkylating agent. But, chemosensitivity to TMZ strongly depends on epigenetic silencing by methylation of the O(6)-Methylguanine-DNA methyltransferase (*MGMT*) promoter [15]. Different randomized trials have shown that methylation of the *MGMT* promoter in GBM patients is associated with significantly higher survival rates if treated with radiotherapy and TMZ [4]. At the stage of recurrent disease, a TMZ rechallenge seems only reasonable in patients with clear methylation of the *MGMT* promoter based on the results of the DIRECTOR trial [24]. Recent data from the NOA-09 trial showed that newly diagnosed GBM patients with methylated *MGMT* promoter might benefit from a more intense first-line treatment regimen with CCNU in combination with TMZ [8], accepting an increased toxicity for an improved prognosis. These trials emphasize the importance of reliable *MGMT* status assessment and the need for predictive cutoff levels for clinical decision-making.

The methylation status of the *MGMT* promoter is widely determined by quantitative pyrosequencing (PSQ) [12, 28]. PSQ analysis uses a defined cutoff value to classify cases as “methylated” or “unmethylated” [1]. In many neurooncological centers, the biological cutoff is 10% [27]. However, a very strict cutoff value might not fully reflect the clinical response to TMZ therapy. Various previous studies that focused on the technical assessment of the *MGMT* status have suggested higher predictive cutoff levels above 10% [14, 17, 18].

Here, we aimed to determine a predictive cutoff level for clinical decision-making on the basis of a well-defined patient cohort of 111 *IDH* wildtype GBM patients. Three methylation groups were identified, which showed a very distinct clinical course in terms of PFS and OS: unmethylated 0-9% (UM), low methylated 10-20% (LM), and highly methylated > 20% (HM).

## Methods and Material

### Tissue samples, clinical and patient data

Two hundred ninety patients with newly diagnosed, previously untreated GBM (WHO grade IV) patients have been diagnosed between 2010 and 2015 at the Departments of Neurosurgery and Neuropathology, Charité Berlin, Germany. GBM diagnosis was confirmed by at least two experienced neuropathologists after surgical resection or stereotactic biopsy. According to the current WHO classification of CNS tumors [13], *IDH* mutation status was determined by IDH1 R132H immunohistochemistry (IHC) and bidirectional Sanger sequencing of exon 4 of the *IDH1* as well as *IDH2* gene for all GBM patients younger than 55 years [13]. Gliosarcoma, epithelioid glioblastoma, giant cell glioblastoma and *IDH* mutant tumors were excluded. The following clinical data were assessed: age at diagnosis, Karnofsky performance status (KPS), tumor localization, extent of resection and residual tumor volume, type and timing of adjuvant therapy, second-line therapy at recurrence, follow-up time, progression-free (PFS) and overall survival (OS) in months. The extent of tumor resection was determined by measuring the contrast-enhancing tumor volume in mm^3^ on T1-subtraction MRI imaging pre- and 48 hours postoperatively using the Brainlab iMRI software (Brainlab AG, Munich, Germany). Gross total resection (GTR) was defined as residual tumor volume < 2% [22]. PFS was assessed according to RANO criteria [25]. We identified 205 *IDH* wildtype GBM patients who matched the criteria mentioned above. Three long-term survivors (LTS; OS > 5 years) were identified in our cohort. For two LTS cases, DNA was sufficient to perform a genome-wide methylation analysis (EPIC array) which confirmed the diagnosis of GBM, *IDH* wildtype (Additional file 1: Figure S3 and Figure S4).

### Ethical statement

This study was conducted according to the ethical principles of medical research involving human subjects according to the Declaration of Helsinki. The clinical data were assessed and anonymized for patients’ confidentiality. Ethical approval (EA2/064/17) was granted by the institutional ethics board of the Charité Ethics Committee.

### DNA extraction, bisulfite treatment and analysis of *MGMT* promoter methylation status in tumor samples

Areas of high tumor cell content (≥ 80%) were chosen and macro-dissected for further analysis (Additional file 1: Figures S1a, dashed line; 1 b). Genomic DNA was extracted from formalin-fixed and paraffin embedded (FFPE) samples using the Qiagen DNeasy blood and tissue DNA extraction kit according to the manufacturer´s protocol (Qiagen, Hilden, Germany). The DNA was sodium bisulfite-modified using the EZ DNA Methylation-Gold™ Kit (Zymo Research, Irvine, CA).

#### Pyrosequencing (PSQ)

Quantitative methylation analyzes were performed using the PyroMark Q24 MGMT kit (Qiagen, Hilden, Germany) and an automated PyroMark Q24 System (Qiagen, Hilden, Germany) following the manufacturer’s instructions. Data was analyzed with the PyroMark Q24 Software 2.0 (Qiagen, Hilden, Germany). The percentage of methylated alleles was calculated as the mean value of the methylation percentage obtained. The cutoff value ≥ 10% was defined to classify *MGMT* methylated vs. unmethylated cases, which is commonly used and has been validated for routine clinical diagnostics [27]. Standardized positive and negative controls were included in every PSQ run. The PSQ results were evaluated by at least two experienced neuropathologists.

#### *Semi-quantitative* methylation-specific PCR (sqMSP)

sqMSP was performed with primers specific for either “methylated” or “unmethylated” DNA as previously described [5]. Original MSP PCR gels are shown in Additional file 1: Figure S2. Primers and PCR programs are listed in the methods and material section of  Additional file 1. Semi-quantitative analysis of the optical band intensity (I) was performed using ImageJ (National Institutes of Health, Bethesda, USA). The following equation was used:

$$ \mathrm{Band}\ \mathrm{in}\mathrm{tensity}\ \mathrm{methylated}\ \mathrm{in}\%=\left(\left({\mathrm{I}}_{\mathrm{meth}}+{\mathrm{I}}_{\mathrm{unmeth}}\right)/{\mathrm{I}}_{\mathrm{meth}}\right)\times 100 $$

$$ \mathrm{Band}\ \mathrm{in}\mathrm{tensity}\ \mathrm{unmethylated}\ \mathrm{in}\%=\left(\left({\mathrm{I}}_{\mathrm{meth}}+{\mathrm{I}}_{\mathrm{unmeth}}\right)/{\mathrm{I}}_{\mathrm{unmeth}}\right)\times 100 $$

#### Direct Bisulfite Sequencing (dBiSeq)

dBiseq was carried out as previously described [16] with minor adaptations. Primers and PCR program are listed in the methods and material section of  Additional file 1. Sequencing was performed at Eurofins Genomics, Ebersberg, Germany. Sequenced samples were returned as .ab1 files, which were then analyzed using Chromas [9] (software program for PC, available at *http://www.technelysium.com.au/chromas.html*).

### Analysis of *MGMT* promoter methylation status in positive and negative controls

Both, positive and negative controls (listed in Additional file 1: Table S1) were assessed by PSQ, sqMSP, and dBiseq. Samples of non-neoplastic brain tissue and one samples with genomic DNA extracted from whole peripheral blood served as negative controls. The primary cell line SF126 and 7 tumor samples with clear *MGMT* promoter methylation levels > 30% were used as positive controls.

### Genome-wide DNA methylation analysis

DNA methylation signature analysis was performed using the Illumina Infinium Methylation EPIC array as previously described [2].

### *IDH1* and *IDH2* Sanger sequencing

Bidirectional Sanger sequencing of exon 4 of *IDH1* and *IDH2* was performed in IDH R132H IHC-negative or -equivocal cases in all patients < 55 years of age. PCR primers for the genomic regions corresponding to *IDH1* exon 4 (codon R132) and *IDH2* exon 4 (codon R172) and the flanking intronic sequences are displayed in the methods and material section of Additional file1. Sequencing was performed at Eurofins Genomics, Ebersberg, Germany.

### Immunohistochemical procedures

Immunohistochemical stainings were performed on a Benchmark XT autostainer (Ventana Medical Systems, Tuscon, AZ, USA) with standard antigen retrieval methods (CC1 buffer, pH8.0, Ventana Medical Systems, Tuscon, AZ, USA) using 4-μm-thick, FFPE tissue sections (Additional file 1: Figures S1 c-f). The following primary antibodies were used: polyclonal rabbit anti-GFAP (1:2000, Dako), monoclonal mouse anti-MIB1 (Ki-67, 1:100, clone M7240, Dako), polyconal rabbit anti-ATRX (1:200, Sigma), mouse monoclonal anti-IDH1 R132H (1:20, clone H09, Dianova). The iVIEW DAB Detection Kit (Ventana Medical Systems, Tuscon, AZ, USA) was used according to the manufacturer's instructions. Sections were counterstained with hematoxylin, dehydrated in graded alcohol and xylene, mounted and coverslipped. IHC stained sections were evaluated by two independent, experienced neuropathologists. When no agreement was reached, the sections were reviewed by our team of neuropathologists at our department (Charité) and further molecular diagnostics (e.g. *IDH1*/*IDH2* bidirectional Sanger sequencing, genome-wide DNA methylation analysis (EPIC analysis)) was performed.

### Statistical analysis

Statistical analysis was performed in cooperation with the Charité´s Institute for Biometrics and Clinical Epidemiology using GraphPad Prism 5 (GraphPad Software, La Jolla, CA, USA). Kaplan-Meier survival curves were obtained and differences in PFS and OS were tested for statistical significance using the log-rank test. Significance level was set at *p* < 0.05.

ROC analysis was used for diagnostic test evaluation. The true positive rate (Sensitivity) was plotted as a function of the false positive rate (100-Specificity) for different cutoff points. The area under the ROC curve (AUC) measured the accuracy. An AUC of 1 represents a perfect test; 0.8-0.9 a good test, 0.7-0.8 a fair test, 0.6-0.7 a poor test, and an area of ≤ 0.5 represents a worthless test.

## Results

### Study cohort

Heterogeneity of the patient cohort (e.g. in terms of the *IDH* status) has been a major point of criticism in previous studies where the predictive mean *MGMT* promoter methylation cutoff had to be determined. Therefore, we selected a homogeneous group of *IDH* wildtype GBM patients with KPS > 70%, who received i) GTR of GBM manifestation, ii) Stupp regime within 4-6 weeks after initial surgery [20], and iii) completed Stupp regime after 6 cycles or until progression of disease, assessed according to the RANO criteria (n=111). All clinical information is displayed in Table 1. GBM diagnosis was confirmed by at least two experienced neuropathologists using a standardized panel of conventional and immunohistochemical stainings (Additional file 1: Figures S1 a-f). All cases were proven *IDH* wildtype by bidirectional Sanger sequencing. Patients with *IDH1* (Additional file 1: Figures S1 g-j) and *IDH2* (Additional file 1: Figure S5) mutant tumors were excluded.

**Table 1:** Patients’ characteristics of our study cohort

Study cohort *n*=111		n	%
Gender	Female	48	43
	Male	63	57
Age in years	Mean	58.9	-
	Median	61.2	-
	Range	18-85.4	-
MGMT	Meth. (mean ≥10%)	56	51
	Unmeth. (mean < 10%)	55	49
Toxicity during 1^st^ line therapy	CTG °III-IV	4	4
2^nd^ line therapy	mTMZ	38	34
	TMZ rechallenge	7	6
	CCNU+Procarbazine	4	4
	BEV	3	3
	Re-irradiation	4	4
	TTFields	5	5
	Re-resection	33	30
Follow up in months	Mean	19.4	-
	Median	15.4	-
	Range	0.3-90	-
	Lost to follow up	2	2
PFS in months	Mean	12	-
	Median	7.8	-
	Range	0.3-56	-
OS in months	Mean	19.8	-
	Median	15.5	-
	Range	0.5-90	-

*BEV* bevacizumab, *CCNU* lomustin, *CTG* common toxicity criteria, *GTR* gross total resection, *MGMT* O^6^-methylguanine-DNA-methyltransferase, *meth* methylated, *mTMZ* metronomic temozolomide, *OS* overall survival, *PR* partial resection, *TMZ* temozolomide, *TTFields* tumor treating fields, *unmeth * unmethylated

**Fig. 1:**
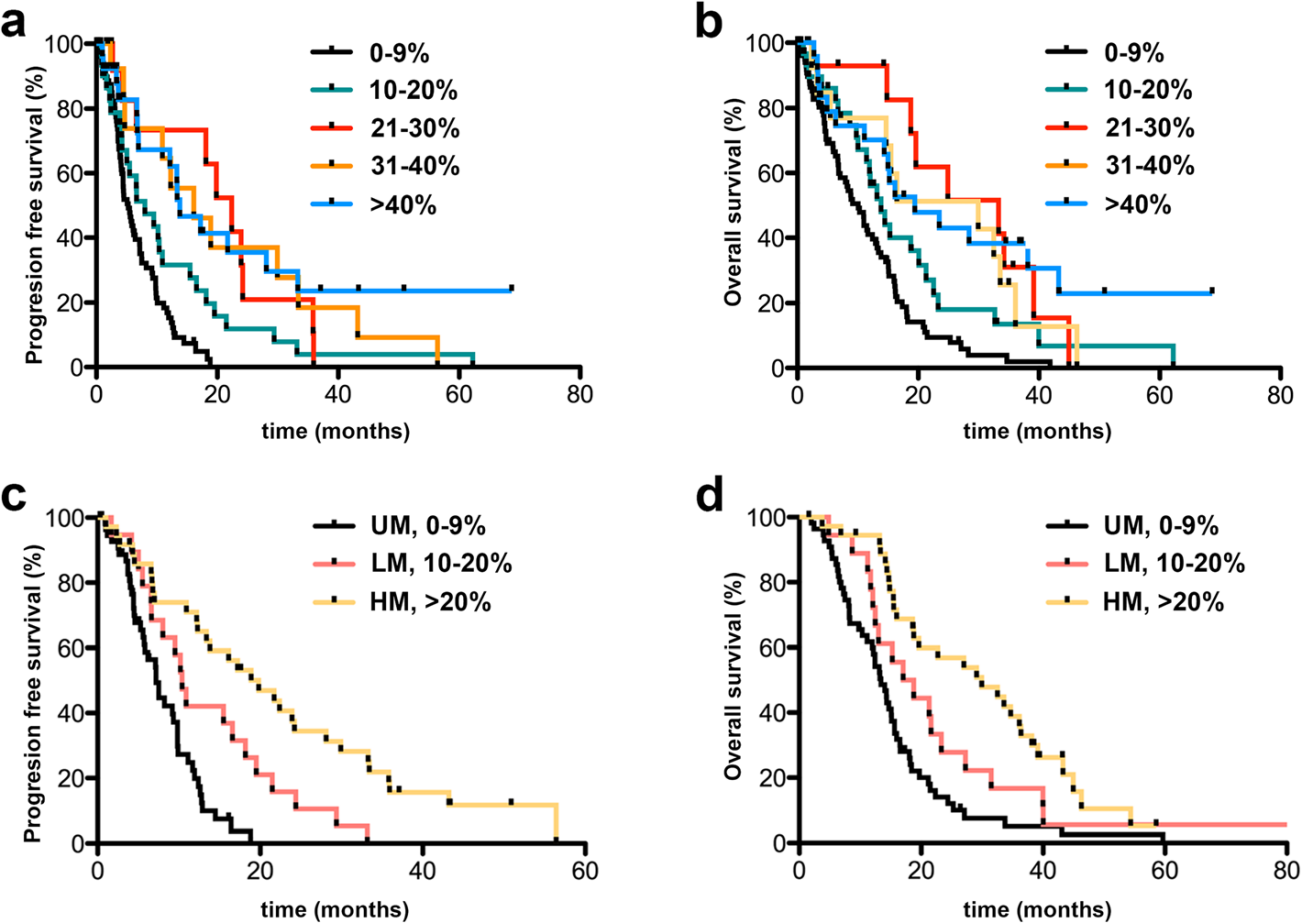
**a**, **b**: Kaplan-Meier curves for progression-free (PFS) and overall survival (OS) of subgroup analysis comparing the different methylation groups (mean *MGMT* promoter methylation): 0-9%, 10-20%, 21-30%, 31-40%, and > 40%. **c**, **d**: Kaplan-Meier curves for progression-free (PFS) and overall survival (OS) of subgroup analysis comparing the different methylation groups UM, LM, and HM according to mean *MGMT* methylation PSQ results

Initially, Kaplan-Meier curves were generated for the following methylation groups (mean *MGMT* promoter methylation): 0-9%, 10-20%, 21-30%, 31-40%, and > 40% (Figure 1 a, b). mPFS in months was 5.28 (0-9%), 8.03 (10-20%), 22.4 (21-30%), 16.13 (31-40%), and 13.8 (> 40%). mOS in months was 10.07 (0-9%), 13.83 (10-20%), 33.33 (21-30%), 29.93 (31-40%), and 19.43 (> 40%). For PFS, Kaplan-Meier curves comparison revealed significant differences between the following groups: 0-9% vs. 10-20% (*p=0.0143, HR 1.745, CI 1.118 to 2.725), 0-9% vs. 21-30% (***p<0.0001, HR 3.307, CI1.885 to 5.800; 0-9% vs. 31-40%), 0-9% vs. 31-40% (***p=0.0002, HR 2.788, CI 1.614 to 4.817), 0-9% vs. > 40% (***p<0.0001, HR 2.869, CI 1.787 to 4.608), and 10-20% vs. > 40% (*p=0.0189, HR 2.109, CI 1.131 to 3.933). For OS, Kaplan-Meier curves comparison demonstrated significant differences between: 0-9% vs. 10-20% (*p=0.0239, HR 1.636, CI1.067 to 2.509), 0-9% vs. 21-30% (***p=0.0003, HR 2.638, CI 1.569 to 4.435), 0-9% vs. 31-40% (**p=0.024, HR 2.252, CI1.332 to 3.805), and 0-9% vs. > 40% (***p<0.0001, HR 2.478, CI 1.565 to 3.922). Since PFS and OS were not significantly different in 21-30%, 31-40%, and > 40%, these groups were combined to one group (> 20%). A survival curve comparison indicated a highly significant difference between 0-9% and > 20% mean *MGMT* methylation in terms of PFS and OS. Consequently, we introduced three major methylation groups: unmethylated 0-9% (UM), low methylated 10-20% (LM) and highly methylated > 20% (HM, Figure 1 c, d). mPFS was 7.2 months in the UM group, 10.4 months in the LM group and 19.83 months in the HM group. Kaplan-Meier curve comparison revealed significant differences between UM vs. LM (**p= 0.0046, HR 2.225, CI 1.280 to 3.869), LM vs. HM (*p=0.0104, HR 4.224, CI 2.443 to 7.303), and UM vs. HM (***p< 0.0001, HR 2.439, CI 1.233 to 4.826). mOS was 13.4 months in the UM group vs. 17.9 months in the LM group vs. 29.93 months in the HM group. Survival differences were not significant for UM vs. LM (p=NS, HR 1.619, CI 0.9780 to 2.680) and for LM vs. HM (p=NS, HR 1.619, CI 0.9780 to 2.680), which was due to one LTS patients within the LM group. OS was significantly different between UM vs. HM (***p< 0.0001, HR 2.900, CI 1.816 to 4.630).

### Defining a transition zone

**Fig. 2:**
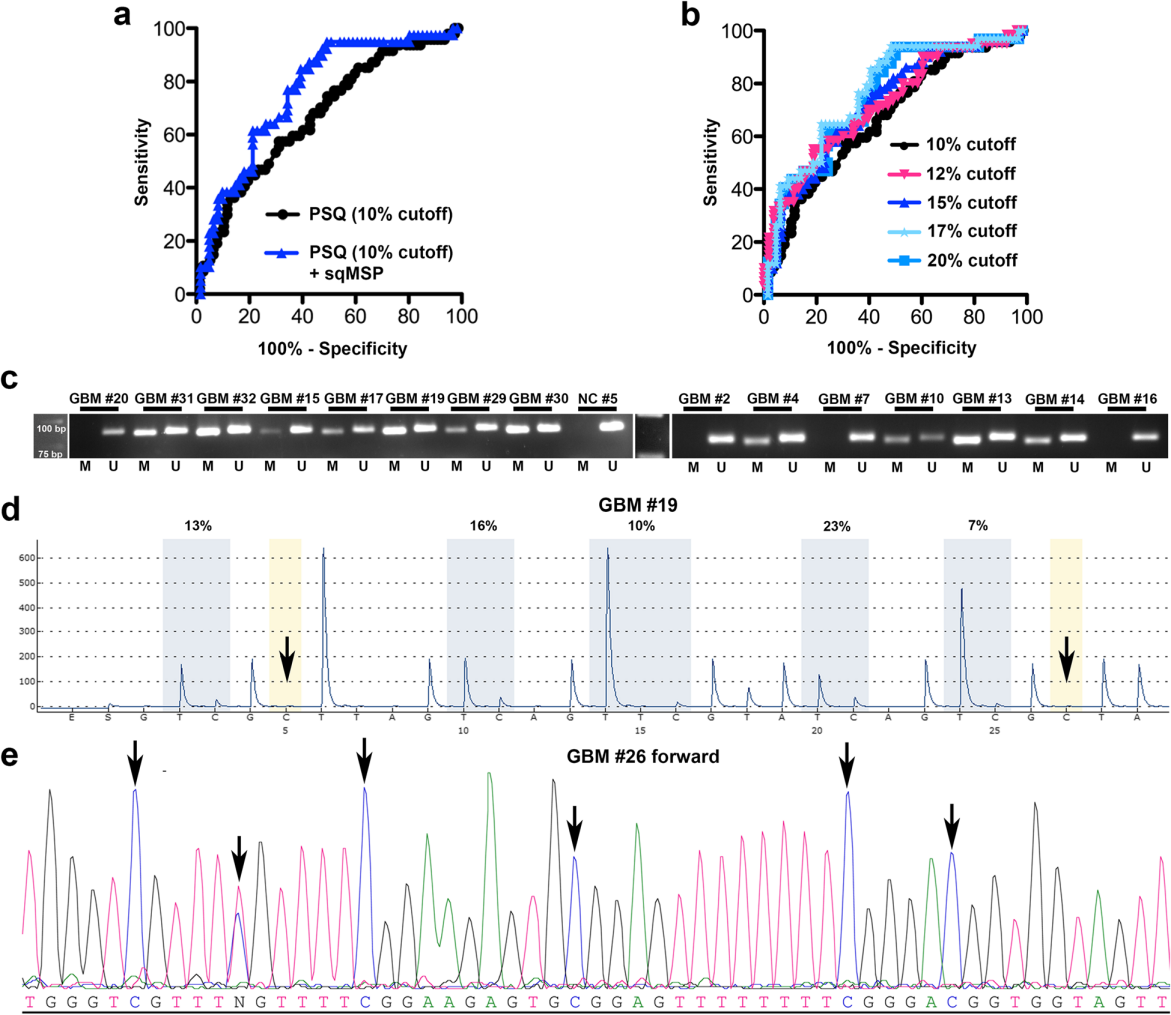
**a**: Receiver operating characteristics (ROC) for 10% PSQ cutoff (AUC = 0.67) and 10% PSQ cutoff corrected for sqMSP results (AUC = 0.76) in terms of overall survival. **b**: Receiver operating characteristics (ROC) for determination of optimal cutoff values for PSQ results at 10% (AUC = 0.67), 12% (AUC = 0.72), 15% (AUC = 0.74), 17% (AUC = 0.77) and 20% (AUC = 0.75). **c**: Representative methylation-specific PCR (MSP) shows results obtained from 15 different, representative GBM samples and one negative control (NC). The DNA was extracted from formalin-fixed and paraffin-embedded tissue. Methylated samples demonstrated PCR products with primers detecting the methylated (M, 89bp) and unmethylated (U, 93bp) *MGMT* promoter sequence. Clearly unmethylated samples showed PCR products only for the unmethylated *MGMT* promoter sequence (U). The pyrogram (**d**) demonstrates the pyrosequencing result of patient #19 (PSQ mean = 14%). The yellow areas correspond to the internal control of conversion (arrows). The blue areas indicate the polymorphism (T/C) as result of the bisulfite treatment and show the level of the methylation (%) of each CpG. An exemplary section of direct Bisulfite Sanger sequencing trace (forward) of patient #26 demonstrates CpGs 13-18 of 27 (arrows) which are partly (T/C) or fully converted (**e**)

LM patients demonstrated a similar clinical course compared to UM patients in terms of PFS and OS, which indicated that the widely used PSQ cutoff of 10% does not fully reflect the clinical response to alkylating agents. We have therefore defined the LM group (10-20%) as a “transition zone” between unmethylated and clearly methylated cases. To validate the PSQ *MGMT* results in this particular subgroup of the unselected study cohort, these cases (LM, n=35) were additionally analyzed by sqMSP (n=32/35). In 53.1 % (n=17/32) sqMSP and PSQ results were disconcordant (representative MSP and PSQ results are shown in Figures 2 c, d). For n=22/35 cases, additional dBiseq data was available (representative results in Fig. 2e). PSQ and dBiseq showed identical results in only 45.5% (n=10/22), MSP and dBiseq in 90% (n=18/20) of cases. The detailed results are displayed in Additional file 1: Table S1. In general, in cases with PSQ ≥ 16%, we observed a very high consistency between PSQ, MSP and dBiseq results.

We additionally investigated the survival profiles of all transition zone patients after combining PSQ and MSP results. First, we redistributed the LM patients to either the UM or HM category based on MSP testing. As expected, the differences between UM vs. HM were highly significant: PFS (***p<0.0001, HR 3.002, CI 1.886 to 4.778) and OS (***p<0.0001, HR 2.629, CI 1.729 to 3.997, Additional file 1: Figure S6 a, b). Next, we defined the following more detailed four groups to investigate if the integration of MSP resulted in a redistribution of LM patients to either the UM or HM category: UM, LM + MSP unmethylated, LM + MSP methylated, and HM. The results still clearly indicated a transition zone for median PFS and OS, which seemed independent of the MSP results (Additional file 1: Figure S6 c, d). Moreover, curve comparison between PSQ LM + MSP unmethylated and PSQ LM + MSP methylated showed no significant difference, most likely due to small sample size and presence of one LTS patients within the LM group.

Regarding the aforementioned results, we performed ROC curve analysis for prognostic test evaluation for PSQ (cutoff 10%) alone and for PSQ (cutoff 10%) combined with sqMSP results. LM cases that were considered *MGMT* unmethylated by sqMSP were therefore assigned to the UM group, LM cases that were considered *MGMT* methylated by sqMSP were therefore assigned to the HM group. ROC curve analysis revealed superior test precision with an AUC = 0.76 for PSQ (cutoff 10%) combined with sqMSP results compared to PSQ (cutoff 10%) alone (AUC = 0.67; Figure 2 a). Additionally, we performed step-wise cutoff testing for 10%, 12%, 15%, 17%, and 20% PSQ results. At a cutoff of 17%, highest test precision was reached with an AUC of 0.77 (Figure 2 b).

## Discussion

We demonstrate that *IDH* wildtype GBM patients with low methylation of the *MGMT* promoter (mean 10-20%) represent a “transition zone” in terms of PFS and OS compared to clearly unmethylated (0-9%) and highly methylated (> 20%) patients. For patients with low methylated *MGMT* promoter (10-20%), PSQ results could be validated in only 51.5 % (n=17/33 samples, Additional file 1: Table S1) by one other method (sqMSP or dBiseq) to be clearly methylated.

Both, MSP and PSQ, have independently been suggested as the “gold standard” for methylation analysis of the *MGMT* gene promoter [3, 11]. As to which method to use, the scientific community has not reached a consensus yet [3, 19]. Several studies have demonstrated the prognostic value of MSP. Nevertheless, MSP primers are generated to detect either unmethylated or fully methylated *MGMT* promoter sites, which may in turn result in a lower sensitivity of this method [10]. Furthermore, MSP lacks international standardization [19]. In contrast to MSP, PSQ provides information about the extent of methylation at each individual CpG site, which improves the sensitivity of analyzing heterogeneous methylation patterns within a tumor sample [10]. Nevertheless, the optimal cutoff value is still a matter of scientific debate [1]. The predictive cutoff is strongly influenced by i) interlaboratory differences, ii) technical challenges of *MGMT* testing, which are strongly dependent on successful bisulfite treatment of the DNA [6], and particularly iii) tissue processing, such as formalin-fixation and paraffin-embedding [17, 18]. Therefore, determining a “grey zone” seems to be a more reasonable approach than setting a very strict cutoff.

Even though previous studies have identified 10% as the PSQ cutoff to distinguish methylated from unmethylated samples - often based on biological determinants comparing non-neoplastic to neoplastic tissue [4, 17, 27, 28] - several more recent studies have suggested to introduce a “transition” or “grey zone” [7, 17, 18, 26] for partly methylated tumors that perhaps cannot be assigned to either the methylated or unmethylated category. Many of these studies were criticized due to small sample size and heterogeneous patient population [28] including different therapeutic regimens and *IDH* mutant as well as *IDH* wildtype GBM patients.

Seeing that *IDH* mutant GBMs demonstrate a hypermethylator phenotype and show a favorable clinical course, the impact of *MGMT* methylation on survival may have been overestimated in those studies [23].

Clearly, our study also has some limitations that restrict the interpretation of our data. There are both, the retrospective character and the single center experience. Nevertheless, a key advantage of this study is that it provides a large data set (n=111) from a both clinically and molecularly very well-documented and characterized subgroup of *IDH* wildtype GBM patients (according to the most recent WHO classification).

As the different methylation groups demonstrate a very distinct clinical course in terms of PFS and OS, and PSQ and sqMSP/sBiseq results are only concordant in 51.5% of LM patients - which might partly be explained by a heterogeneous methylation pattern and technique-dependent analysis of different CpG sites within the MGMT promoter [19] - we conclude that PSQ results in patients with low *MGMT* promoter methylation (10-20%) should be interpreted with caution. If therapeutically relevant, a second technique, e.g. MSP could be additionally used to substantiate the results in *MGMT* PSQ transitional (10-20%) cases. Our ROC curve analysis indicates that the combination of PSQ and MSP results is diagnostically beneficial in the LM patient cohort. Our results, furthermore, suggest 17% as the most accurate cutoff value for PSQ analysis. It has been the consensus in clinical practice to also treat patients with low level *MGMT* methylation as a potential benefit cannot be excluded. Nevertheless, further scientific investigation is necessary to establish this efficacy. Especially in elderly (≥ 70 years) or fragile GBM patients, a further stratification would be favorable as these patients have a higher risk of chemotherapy-related toxicity and demonstrate less survival benefit from alkylating agents if *MGMT* is unmethylated [19]. To conclude, we recommend the following classification system be used (particularly if FFPE samples are used): clearly unmethylated (< 10%), low methylated (between 10-20%), and clearly methylated (> 20%), which correlated with significantly improved PFS and OS in our cohort.

## Additional file

Additional file 1: **Figure S1.** Glioblastoma histology and representative *IDH1* Sanger sequencing results. **Figure S2.** Original MSP PCR gels. **Figure S3.** Methylation profiling report of GBM LTS patient #20 (GBM #20). **Figure S4.** Methylation profiling report of GBM LTS patient #14 (GBM #14). **Figure S5.** *IDH2* mutation. **Figure S6.** Kaplan-Meier curves for progression-free (PFS) and overall survival (OS) after combining PSQ and MSP results. **Table S1.** MGMT PSQ result of subgroup LM (10-20%) and corresponding sqMSP and dBiseq results. (DOCX 6001 kb)

## Data Availability

The datasets supporting the conclusions of this article are included within the article and its additional file.

## Abbreviations

CCNU: cyclonexyl-chloroethyl-nitrosourea
dBiSeq: direct bisulfite sequencing
FFPE: formalin-fixed and paraffin embedded
GTR: gross total resection
H&E: hematoxylin and eosin
HM: highly methylated
IDH: isocitrate dehydrogenase
LM: low methylated
meth: methylated
MGMT: O(6)-Methylguanine-DNA methyltransferase
OS: overall survival
PFS: progression-free survival
PSQ: Pyrosequencing
ROC: receiver operating characteristic
sqMSP: semi-quantitative methylation specific PCR
TMZ: Temozolomide
UM: unmethylated
unmeth: unmethylated
