# Supplementary material for: Predictive MGMT status in a homogeneous cohort of IDH wildtype glioblastoma patients
Source: Acta Neuropathol Commun. 2019 Jun 5;7:7. doi: 10.1186/s40478-019-0745-z (PMC6549362; doi:10.1186/s40478-019-0745-z)
Supplement: Supplementary file 1 — Figure S1. Glioblastoma histology and representative IDH1 Sanger sequencing results. Figure S2. Original MSP PCR gels. Figure S3. Methylation profiling report of GBM LTS patient #20 (GBM #20). Figure S4. Methylation profiling report of GBM LTS patient #14 (GBM #14). Figure S5. IDH2 mutation. Figure S6. Kaplan-Meier curves for progression-free (PFS) and overall survival (OS) after combining PSQ and MSP results. Table S1. MGMT PSQ result of subgroup LM (10-20%) and corresponding sqMSP and dBiseq results. (DOCX 6001 kb) [file 40478_2019_745_MOESM1_ESM.docx]

**Supplemental material**

**Supplemental material and methods**

1. Methylation-specific PCR (MSP)

The primers used for Methylation-specific PCR (MSP) were:

MGMT_met_for: GTTTTTAGAACGTTTTGCGTTTCGAC

MGMT_met_rev: CACCGTCCCGAAAAAAAACTCCG

MGMT_unmet_for: TGTGTTTTTAGAATGTTTTGTGTTTTGAT

MGMT_unmet_rev: CTACCACCATCCCAAAAAAAAACTCCA

The PCR conditions were 95°C for 15min (1 cycle), followed by 45 cycles of 95°C for 30 s, 56°C for 30 s, 72°C for 90 s and finally extension at 72°C for 10 min with HotStarTaq polymerase (Qiagen Hilden, Germany).

1. Direct bisulfite sequencing (dBiSeq):

The primers used for bisulfite-treated DNA were:

MGMT_Bis_for: GGATATGTTGGGATAGTT

MGMT_Bis_rev: AAACTAAACAACACCTAAA

The PCR conditions were 95°C for 10 min (1 cycle), followed by 3 cycles of 95°C for 30 s, 55°C for 45 s, 72°C for 60 s, 3 cycles of 95°C for 30 s, 53°C for 45 s, 72°C for 60 s, 3 cycles of 95°C for 30 s, 51°C for 45 s, 72°C for 60 s, 33 cycles of 95°C for 30 s, 48°C for 45 s, 72°C for 60 s, and finally extension at 72°C for 10 min with HotStarTaq polymerase (Qiagen Hilden, Germany).

1. *IDH1* and *IDH2* Sanger sequencing

PCR primers for the genomic region corresponding to IDH1 exon 4, which encodes codon R132, and the flanking intronic sequences were as follows:

IDH1_R132_for: CGGTCTTCAGAGAAGCCATT

IDH1_R132_rev: CACATACAAGTTGGAAATTTCTGG

PCR primers for the genomic region corresponding to IDH2 exon 4, which encodes codon R172, and the flanking intronic sequences were as follows:

IDH2_R172_for: CCAATGGAACTATCCGGAAC

IDH2_R172_rev: CTAGGCGAGGAGCTCCAGT

The PCR conditions were 94°C for 4 min (1 cycle), followed by 3 cycles of 94°C for 30 s, 61°C for 45 s, 72°C for 60 s, 3 cycles of 94°C for 30 s, 59°C for 45 s, 72°C for 60 s, 3 cycles of 94°C for 30 s, 57°C for 45 s, 72°C for 60 s, 31 cycles of 94°C for 30 s, 55°C for 45 s, 72°C for 60 s, and finally extension at 72°C for 10 min with AmpliTaq™ 360 DNA Polymerase (Applied Biosystems). Sequencing was performed at Eurofins Genomics, Ebersberg, Germany.

**Supplemental figures**

Supplemental figure 1: Glioblastoma histology and representative *IDH1* Sanger sequencing results


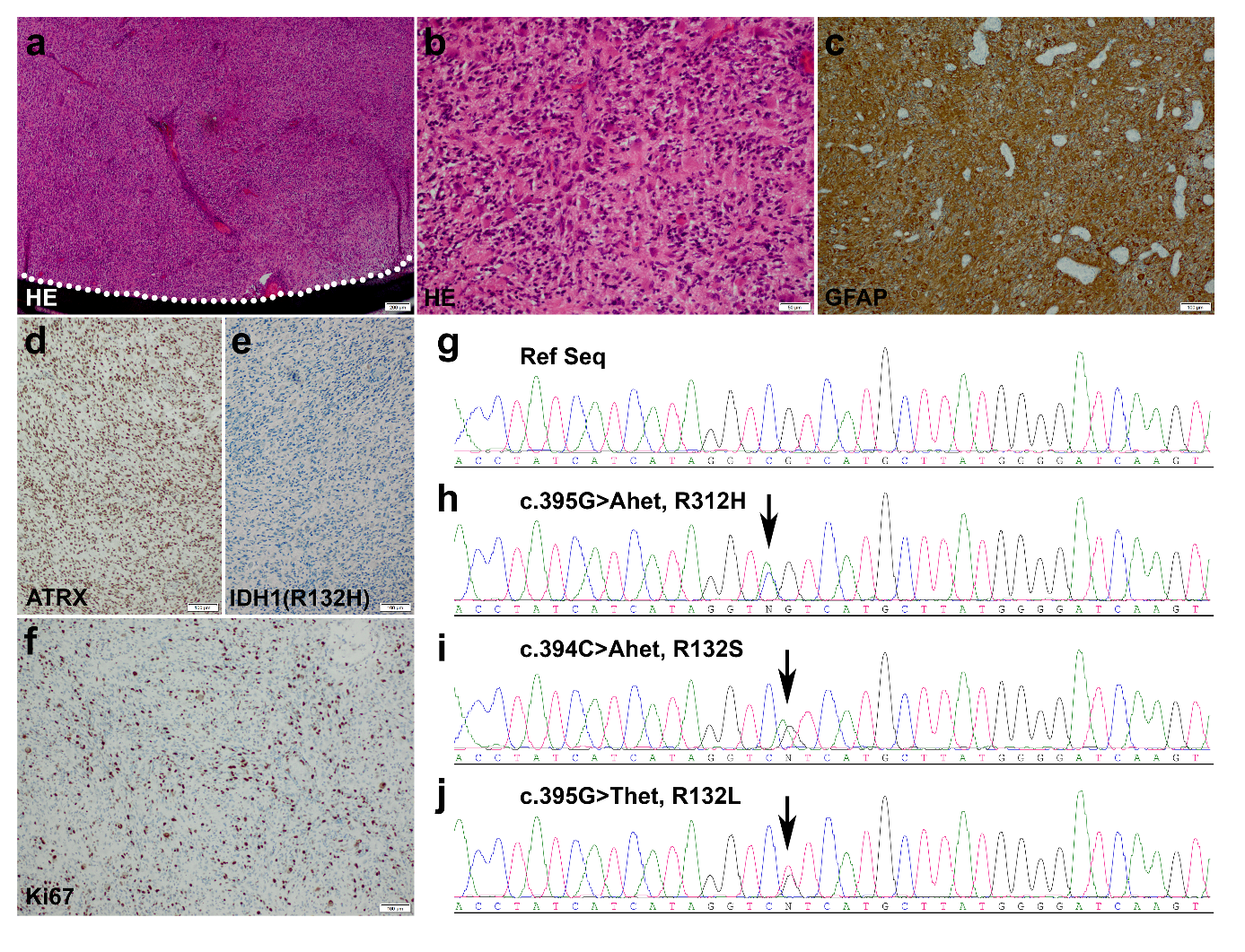


**(a, b)**: H&E staining of a representative GBM tumour sample. Macro-dissection ((**a**); dashed line) ensured high percentage of tumour cells (≥ 80%, (**b**)) for DNA isolation and further analysis. All GBM samples were stained with antibodies against glial fibrillary acidic protein (GFAP; (**c**)), Ki67 (Mib-1; (**d**)), ATRX (**e**), and IDH1 (R132H, (**f**)). **(g-j)**: Representative Sanger sequencing chromatograms presenting the different IDH1 mutations (arrows) found in our patient collective (R132H, R132S, and R132L). Scale bars correspond to: 200 µm (**a**), 50 µm (**b**), 100 µm (**c-f**).

Supplemental figure 2: Original MSP PCR gels

| **100 bp**  **75 bp** | 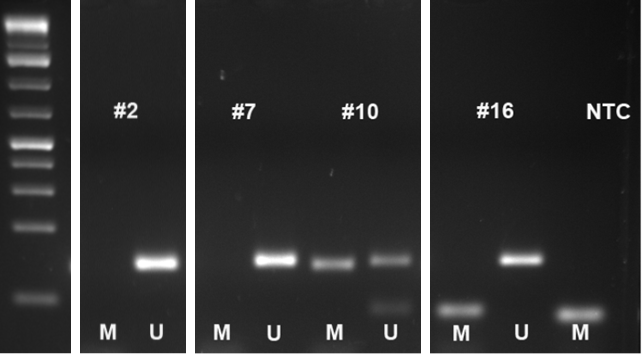 | | | | |
| --- | --- | --- | --- | --- | --- |
| 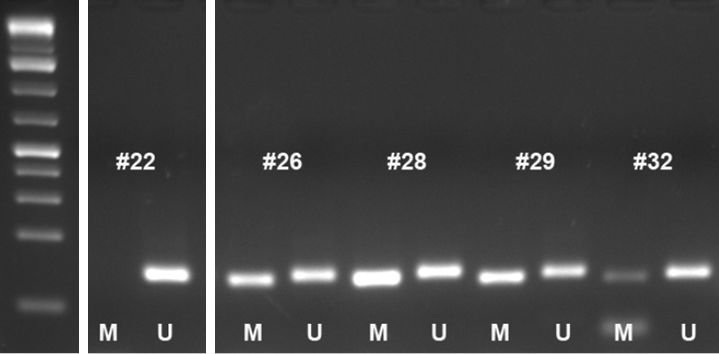 | | | 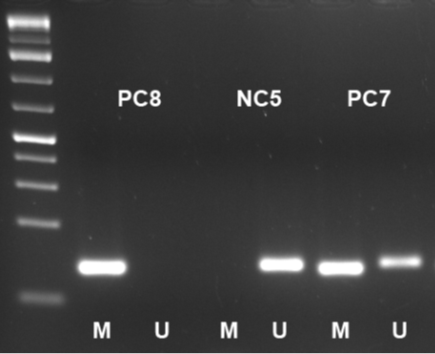 | | |
| 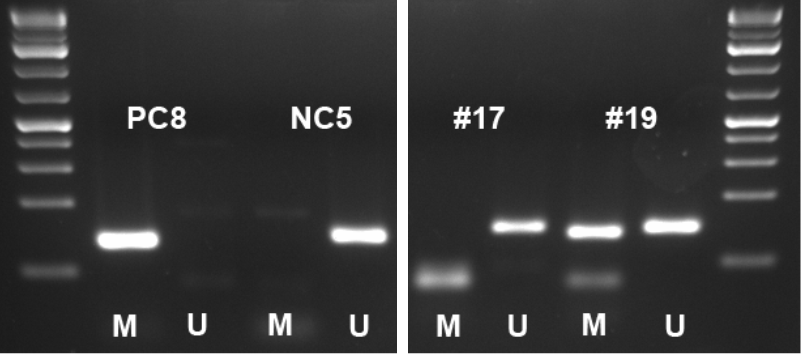 | | | | | 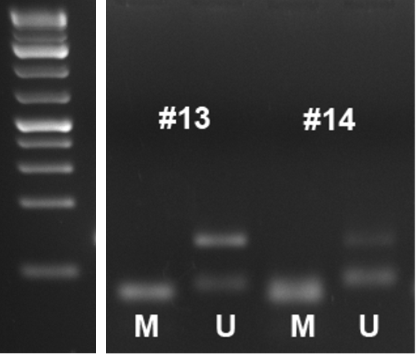 |
| 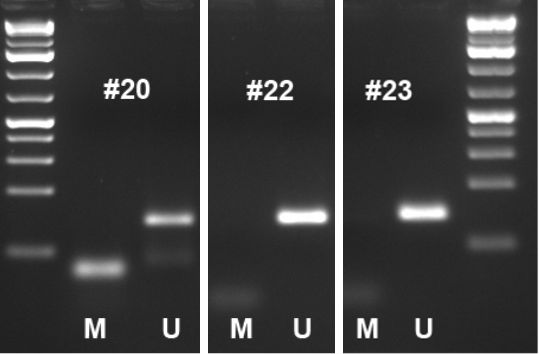 | | 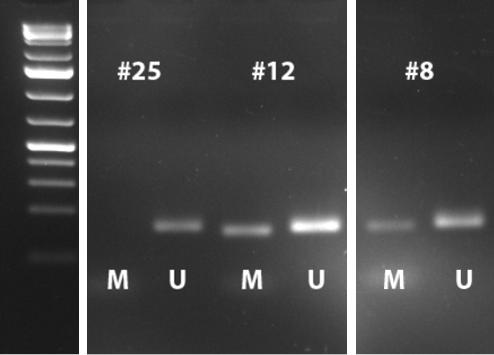 | | | |
| 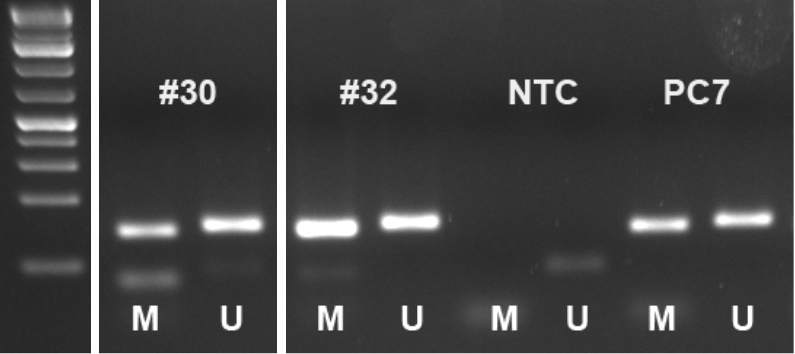 | | | |  | |
|  | | | | | |
| 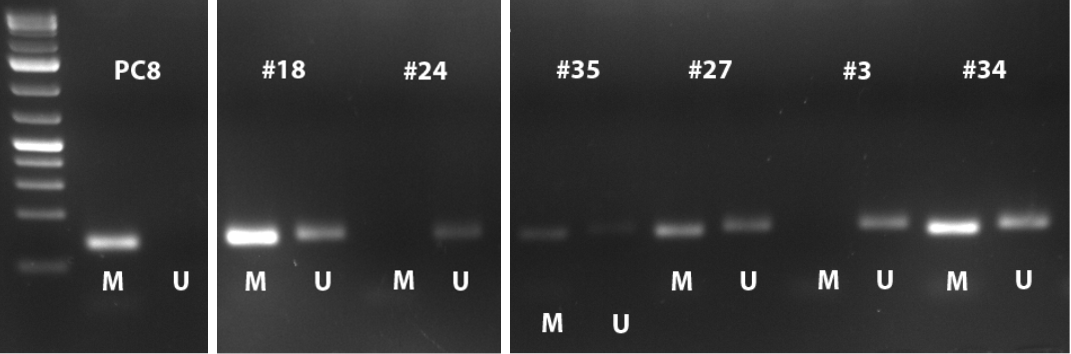 | | | | | |
| 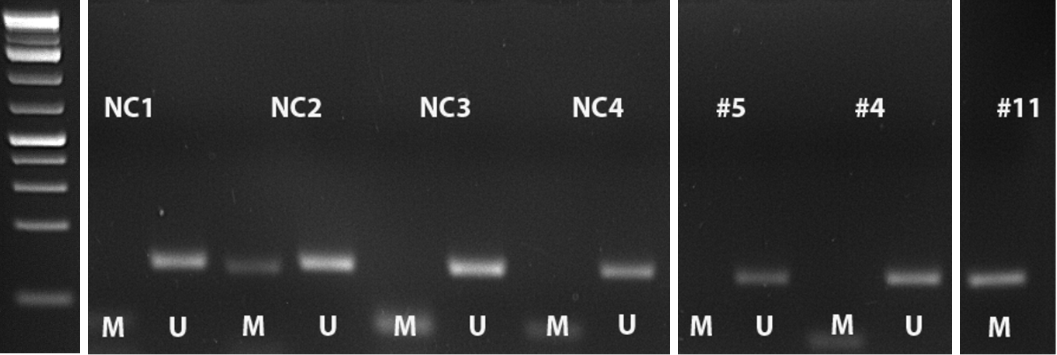 | | | | | |
| PCR gels demonstrate MSP results for all investigated patients as well as positive (PC) and negative controls (NC). NTC: no template control. Methylated samples demonstrated PCR products with primers detecting the methylated (M, 89bp) and unmethylated (U, 93bp) MGMT promoter sequence. Clearly unmethylated samples showed PCR products only for the unmethylated MGMT promoter sequence (U).  Supplemental figure 3: Methylation profiling report of GBM LTS patient #20 (GBM #20)  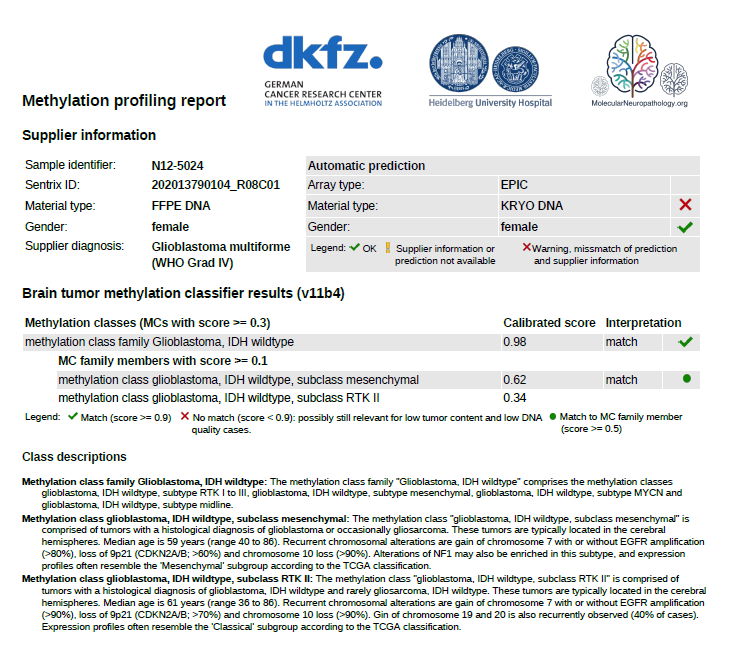 | | | | | |

Supplemental figure 4: Methylation profiling report of GBM LTS patient #14 (GBM #14)

#

Supplemental figure 5: *IDH2* mutation


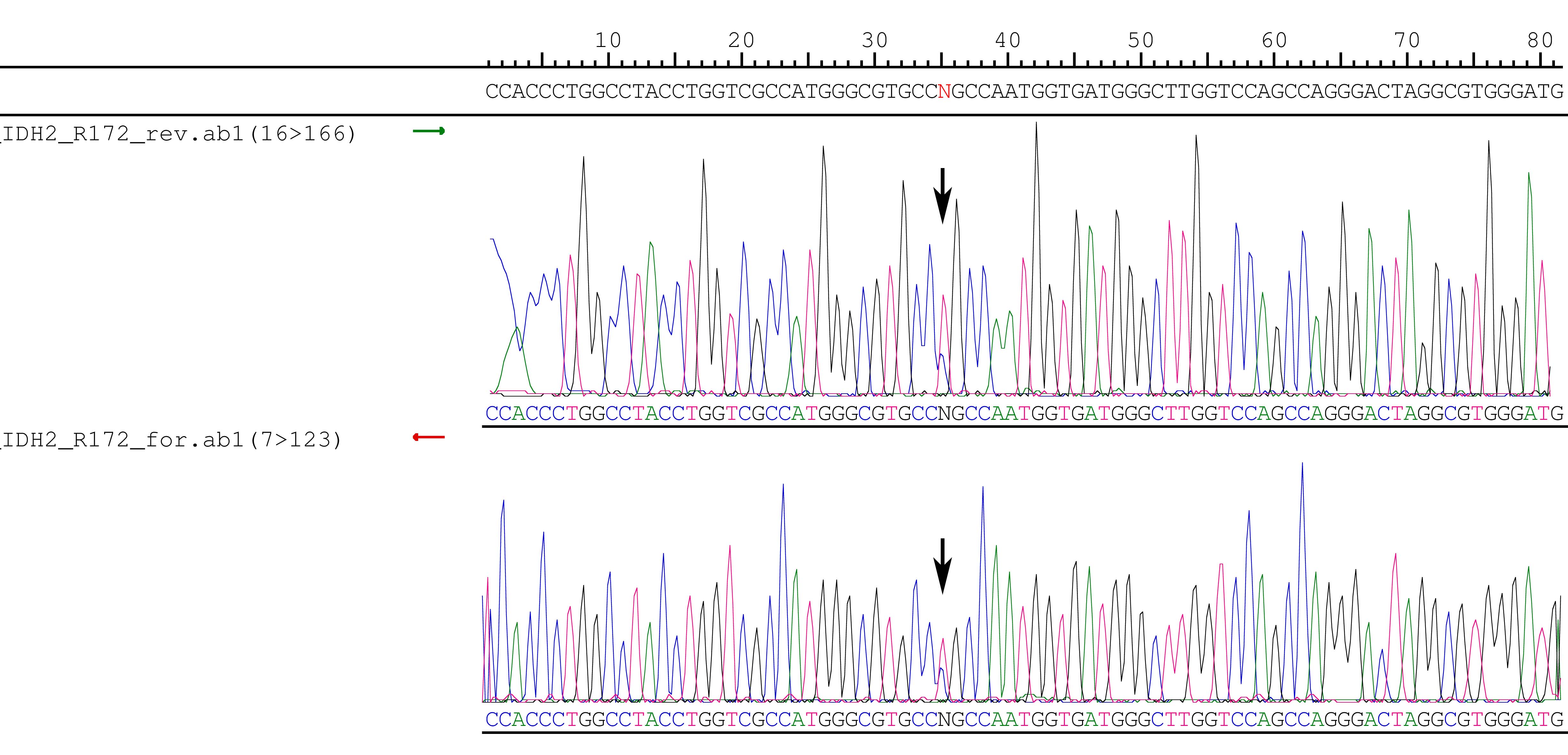


Sanger sequencing chromatogram shows one *IDH2* mutation (arrows) found in our patient collective (c.514A>G, R172G).

Supplemental figure 6: Kaplan-Meier curves for progression-free (PFS) and overall survival (OS) after combining PSQ and MSP results


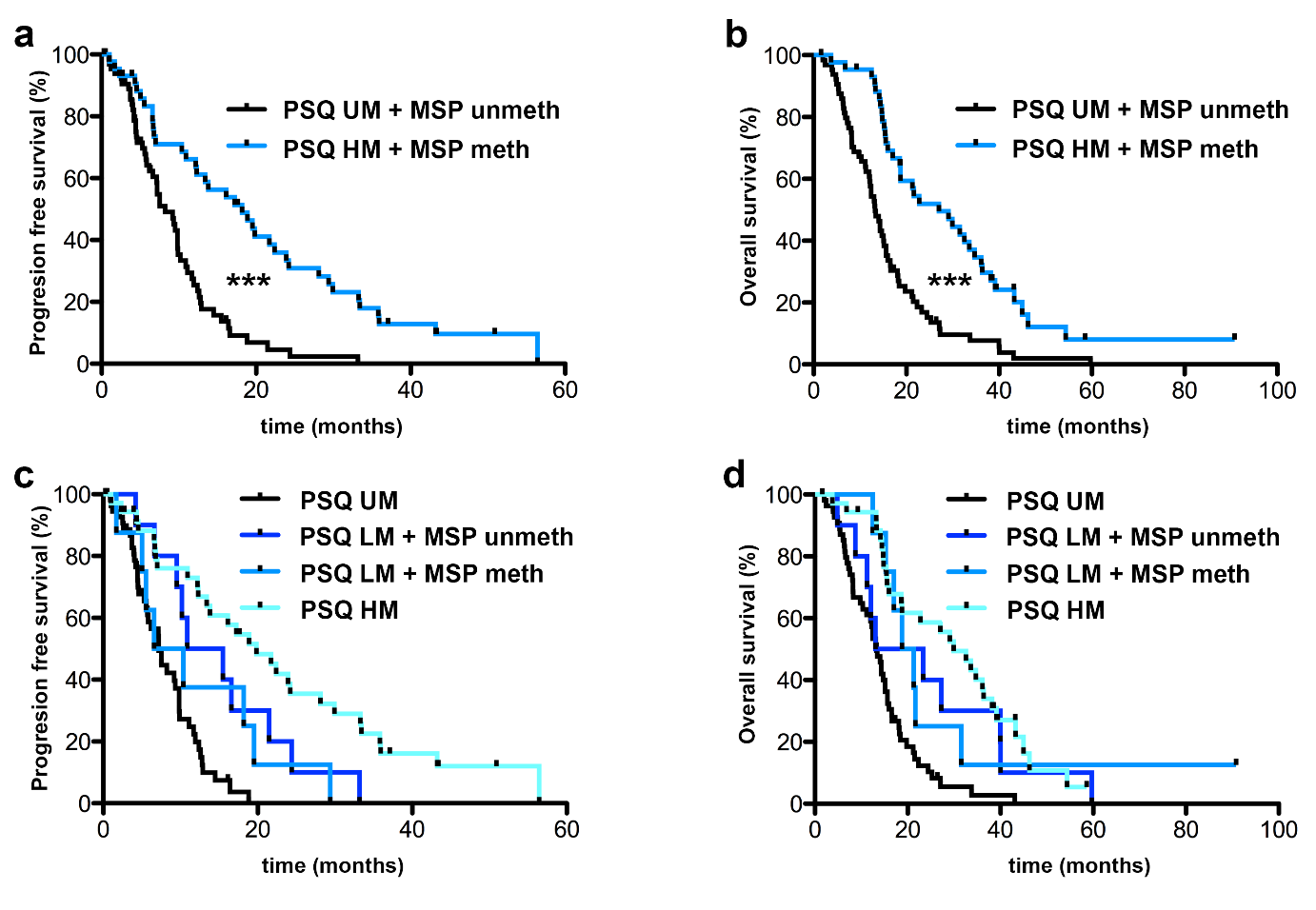


**(a, b)**: Kaplan-Meier curves for progression-free (PFS) and overall survival (OS) showed a highly significant difference (PFS (***p<0.0001, HR 3.002, CI 1.886 to 4.778) and OS (***p<0.0001, HR 2.629, CI 1.729 to 3.997) comparing HM and UM patients after redistribution of the LM patients to either the UM or HM category based on MSP testing. **(c, d)**: Kaplan-Meier curves for progression-free (PFS) and overall survival (OS) of subgroup analysis of the following more detailed four groups combining PSQ and MSP results: UM, LM + MSP unmethylated, LM + MSP methylated, and HM. The results for the LM patients still clearly indicate a transition zone for median PFS and OS.

# Supplemental tables

Supplement table 1: MGMT PSQ result of subgroup LM (10-20%) and corresponding sqMSP and dBiseq results

| **LM subgroup PSQ results (10-20%)** | | | | | | | | | | | | |
| --- | --- | --- | --- | --- | --- | --- | --- | --- | --- | --- | --- | --- |
| **MGMT results** | | | | | | | | | **Match between techniques** | | | |
| **GBM (#)** | **PSQ mean (%)** | | **MSP** | | **MSP**  **semi-quantitative**  **M/U (%)** | | **dBiseq** | **Chro-mas** | **PSQ/**  **MSP** | **PSQ/**  **dBiseq** | **dBiSeq/**  **/MSP** | |
| **1** | 10 | | unclear | | - | | meth. | 39/61 | - | ✓ | - | |
| **2** | 10 | | unmeth. | | 0/100 | | unmeth. | 0/100 | x | x | ✓ | |
| **3** | 10 | | unmeth. | | 0/100 | | UC |  | x | - | - | |
| **4** | 10 | | unmeth. | | 0/100 | | unmeth. | 68/32 | x | x | ✓ | |
| **5** | 10 | | unmeth. | | 0/100 | | UC |  | x | - | - | |
| **6** | 11 | | unclear | | - | | UC |  | - | - | - | |
| **7** | 11 | | unmeth. | | 0/100 | | unmeth. | 0/100 | x | x | ✓ | |
| **8** | 11 | | partially meth. | | 14/86 | | unmeth. | 0/100 | ✓ | x | x | |
| **9** | 12 | | partially meth. | | 40/60 | | meth. | 77/23 | ✓ | ✓ | ✓ | |
| **10** | 12 | | partially meth. | | 39/61 | | unmeth. | 6/94 | ✓ | x | x | |
| **11** | 12 | | partially meth. | | 54/46 | | meth. | 52/48 | ✓ | ✓ | ✓ | |
| **12** | 12 | | partially meth. | | 38/62 | | UC |  | ✓ | - | - | |
| **13** | 13 | | unmeth. | | 0/100 | | unmeth. | 56/44 | x | x | ✓ | |
| **14** | 13 | | unmeth. | | 0/100 | | unmeth. | 65/35 | x | x | ✓ | |
| **15** | 13 | | unmeth. | | 0/100 | | unmeth. | 55/45 | x | x | ✓ | |
| **16** | 13 | | unmeth. | | 0/100 | | UC |  | x | - | - | |
| **17** | 13 | | unmeth. | | 0/100 | | unmeth. | 84/16 | x | x | ✓ | |
| **18** | 14 | | partially meth. | | 79/21 | | meth. | 67/33 | ✓ | ✓ | ✓ | |
| **19** | 14 | | partially meth. | | 56/44 | | meth. | 76/24 | ✓ | ✓ | ✓ | |
| **20** | 14 | | unmeth. | | 0/100 | | UC | UC | x | - | - | |
| **21** | 14 | | NA | |  | | NA |  | - | - | - | |
| **22** | 15 | | unmeth. | | 0/100 | | unmeth. | 1/99 | x | x | ✓ | |
| **23** | 15 | | unmeth. | | 0/100 | | unmeth. | 61/39 | x | x | ✓ | |
| **24** | 16 | | unmeth. | | 0/100 | | unmeth. | 0/100 | x | x | ✓ | |
| **25** | 16 | | unmeth. | | 0/100 | | UC |  | x | - | - | |
| **26** | 16 | | partially meth. | | 50/50 | | meth | 56/44 | ✓ | ✓ | ✓ | |
| **27** | 16 | | partially meth. | | 64/36 | | UC |  | ✓ | - | - | |
| **28** | 17 | | partially meth. | | 56/44 | | UC |  | ✓ | - | - | |
| **29** | 17 | | partially meth. | | 40/60 | | meth. | 57/43 | ✓ | ✓ | ✓ | |
| **30** | 17 | | partially meth. | | 49/51 | | meth. | 72/28 | ✓ | ✓ | ✓ | |
| **31** | 18 | | unclear | | - | | meth. | 40/60 | - | ✓ | - | |
| **32** | 18 | | partially meth. | | 48/52 | | meth. | 61/39 | ✓ | ✓ | ✓ | |
| **33** | 20 | | unmeth. | | 0/100 | | NA |  | x | - | - | |
| **34** | 20 | | partially meth. | | 66/34 | | UC |  | ✓ | - | - | |
| **35** | 20 | | partially meth. | | 81/19 | | UC |  | ✓ | - | - | |
| **Positive controls** | | | | | | | | | | | | |
| **1 (PC1)** | | >60 | | partially meth. | | 85/15 | UC |  | ✓ | - | - | |
| **2 (PC2)** | | >60 | | partially meth. | | 61/39 | meth. | 68/32 | ✓ | ✓ | ✓ | |
| **3 (PC3)** | | >60 | | partially meth. | | 60/40 | meth. | 76/24 | ✓ | ✓ | ✓ | |
| **4 (PC4)** | | >60 | | partially meth. | | 65/35 | meth. | 82/18 | ✓ | ✓ | ✓ | |
| **5 (PC5)** | | >60 | | partially meth. | | 74/26 | UC |  | ✓ | ✓ | ✓ | |
| **6 (PC6)** | | >60 | | partially meth. | | 76/24 | meth. | 54/46 | ✓ | ✓ | ✓ | |
| **7 (PC7)** | | 36 | | partially meth. | | 58/42 | meth. | 78/22 | ✓ | ✓ | ✓ | |
| **8 (PC8)** | | 100 | | meth. | | 100 | meth. | 84/16 | ✓ | ✓ | ✓ | |
| **Negative controls** | | | | | | | | | | | | |
| **1 (NC1)** | | 3 | | unmeth. | | 0/100 | NA |  | ✓ | - | | - |
| **2 (NC2)** | | 3,6 | | partially meth. | | 17/83 | NA |  | x | - | | - |
| **3 (NC3)** | | 5,4 | | unmeth. | | 0/100 | NA |  | ✓ | - | | - |
| **4 (NC4)** | | 5,8 | | unmeth. | | 7/93 | NA |  | ✓ | - | | - |
| **5 (NC5)** | | 2,3 | | unmeth. | | 0/100 | unmeth. | 0/100 | ✓ | ✓ | | ✓ |

✓= match of results between two assays, x = no match of results between two assays, - = not assessed, dBiSeq=direct bisulfite sequencing, MGMT=O^6^methylguanine-DNA-methyltransferase, meth=methylated, unmeth=unmethylated, sqMSP=semi-quantitative methylation specific PCR, PSQ = pyrosequencing, NA=not assessed (no DNA), NC=negative control, PC=positive control, UC=unclear result.
